# Stress-Induced Mucus Secretion and Its Composition by a Combination of Proteomics and Metabolomics of the Jellyfish *Aurelia coerulea*

**DOI:** 10.3390/md16090341

**Published:** 2018-09-18

**Authors:** Wenwen Liu, Fengfeng Mo, Guixian Jiang, Hongyu Liang, Chaoqun Ma, Tong Li, Lulu Zhang, Liyan Xiong, Gian Luigi Mariottini, Jing Zhang, Liang Xiao

**Affiliations:** 1 College of Traditional Chinese Medicine, Jilin Agricultural University, Changchun 130118, China; lww0325@hotmail.com (W.L.); lianghongyu0819@hotmail.com (H.L.); 2 Department of Marine Biotechnology, Faculty of Naval Medicine, Second Military Medical University, Shanghai 200433, China; maria940501@outlook.com (C.M.); zhanglulu2822@outlook.com (L.Z.); 3 Department of Ship Hygiene, Faculty of Navy Medicine, Second Military Medical University, Shanghai 200433, China; fengfengmo@hotmail.com; 4 Clinical Medicine, Grade 2015, Second Military Medical University, Shanghai 200433, China; jiangdaxian@hotmail.com; 5 School of Marine Sciences, Ningbo University, Ningbo 315211, China; 18317128586@163.com; 6 Department of Traditional Chinese Medicine Identification, School of Pharmacy, Second Military Medical University, Shanghai 200433, China; xlyiverson@126.com; 7 Department of Earth, Environment and Life Sciences (DISTAV), University of Genova, Viale Benedetto XV 5, I-16132 Genova, Italy; Gian.Luigi.Mariottini@unige.it

**Keywords:** jellyfish, *Aurelia coerulea*, mucus, proteomics, metabolomics

## Abstract

Background: Jellyfish respond quickly to external stress that stimulates mucus secretion as a defense. Neither the composition of secreted mucus nor the process of secretion are well understood. Methods: *Aurelia coerulea* jellyfish were stimulated by removing them from environmental seawater. Secreted mucus and tissue samples were then collected within 60 min, and analyzed by a combination of proteomics and metabolomics using liquid chromatography coupled with tandem mass spectrometry (LC-MS/MS) and ultra-performance liquid chromatography/quadrupole time-of-flight mass spectrometry (UPLC-QTOF-MS/MS), respectively. Results: Two phases of sample collection displayed a quick decrease in volume, followed by a gradual increase. A total of 2421 and 1208 proteins were identified in tissue homogenate and secreted mucus, respectively. Gene Ontology (GO) analysis showed that the mucus-enriched proteins are mainly located in extracellular or membrane-associated regions, while the tissue-enriched proteins are distributed throughout intracellular compartments. Tryptamine, among 16 different metabolites, increased with the largest-fold change value of 7.8 in mucus, which is consistent with its involvement in the Kyoto Encyclopedia of Genes and Genomes (KEGG) pathway ‘tryptophan metabolism’. We identified 11 metalloproteinases, four serpins, three superoxide dismutases and three complements, and their presence was speculated to be related to self-protective defense. Conclusions: Our results provide a composition profile of proteins and metabolites in stress-induced mucus and tissue homogenate of *A. coerulea.* This provides insight for the ongoing endeavors to discover novel bioactive compounds. The large increase of tryptamine in mucus may indicate a strong stress response when jellyfish were taken out of seawater and the active self-protective components such as enzymes, serpins and complements potentially play a key role in innate immunity of jellyfish.

## 1. Introduction

In recent decades, jellyfish blooms have become an important issue in coastal areas worldwide. These blooms are likely related to issues such as overfishing, global warming and eutrophication [1]. Blooming jellyfish consume fish eggs, crush captured fish, clog or destroy fish nets and block power-plant intakes, leading to disruption of marine ecosystems and thereby causing significant economic losses. Moreover, with massive increases of jellyfish blooms in recent years in coastal areas, the number of victims stung by jellyfish, including swimmers, fishermen and divers, has consequently increased [2,3]. Contact with jellyfish tentacles can trigger millions of nematocysts to pierce the skin and inject venom, causing responses ranging from no effect or local pain, to a series of severe systemic manifestations such as cardiovascular collapse, liver dysfunction, renal failure, and even death [4,5,6]. It is widely reported that the comprehensive toxicities of jellyfish venoms are attributed to numerous active components exerting hemolytic, cardiovascular, muscular, neural, antioxidant and cytotoxic effects [7,8].

An often reported scenario is that children touch or pick up a dying or dead jellyfish while playing on the beach and are stung. The usual speculation is that the poisoning comes from the nematocyst venom released when the tentacles are touched. Interestingly, however, almost all fishermen interviewed described to us that even the ‘residual seawater’ on their fishing nets after trawling through a jellyfish bloom when in contact with their bodies would cause serious cutaneous pain and local swelling. Residual seawater from a jellyfish bloom is reported to be very sticky, most likely derived from a mixture of the secreted mucus [9,10,11,12] and the nematocyst venom of the jellyfish. In the laboratory we have also observed that jellyfish secreted mucus as a defense resulting from external stimulation of being gently shaken or stirred. Moreover, because the gelatinous body of jellyfish is very fragile and easy to autolyze, it is reasonable that a beached jellyfish would also have started to autolyze when touched or picked up by children. Therefore, the envenomation of children by beached jellyfish would likely be due to a mixture of nematocyst venom, secreted mucus and autolyzed tissue fluid.

Like most other aquatic organisms, the surfaces of jellyfish are covered by a thin layer of mucus that originates from the epidermal cells. It is greatly affected when jellyfish are processed or when interfered with [13]. Some species of jellyfish can produce nets of mucus or release blobs to trap food particles [14]. Meanwhile, skin mucus from other aquatic organisms, like fish, contain a variety of immunity-related factors including lectins, lysozymes, calmodulin, immunoglobulins, complement, C-reactive proteins, proteolytic enzymes and anti-microbial peptides [15,16,17]. Although there are few papers reported, it is reasonable to speculate that jellyfish mucus is a rich library of active components for predation by adhesion and digestion, as well as modulators of innate immunity against triggers such as physical damage, microbial invasion and pollutants [14,18,19,20,21].

*Aurelia coerulea* (*Aurelia* sp.1) is a species of moon jellyfish found in the coastal waters of Chinese seas [22,23]. Compared with other marine jellyfish, *A. coerulea* is of low toxicity and can be maturely reproduced in an artificial environment, thus facilitating mucus collection [13]. Our interest in *A. coerulea* is focused on exploring its stress-induced mucus secretion and its composition by a combination of proteomics and metabolomics. Consequently, we aimed to provide insight into the protein and metabolite composition of stress-induced mucus and tissue homogenate to facilitate a better understanding of the process of stress-induced mucus secretion, as well as its involvement in innate immunity, along with the discovery of novel bioactive compounds.

## 2. Results

### 2.1. Stress-Induced Mucus Secretion and Autolysis of A. coerulea

Jellyfish are able to respond quickly to external environmental stimuli, although they have limited movement ability. We have previously noted the active secretion of jellyfish mucus induced by external stimulation, in that the surrounding seawater turns sticky when disturbed. In this study, we first checked the quantity-time relationship of stress-induced mucus secretion as well as the autolysis that rapidly occurs in dying jellyfish.

**Figure 1:**
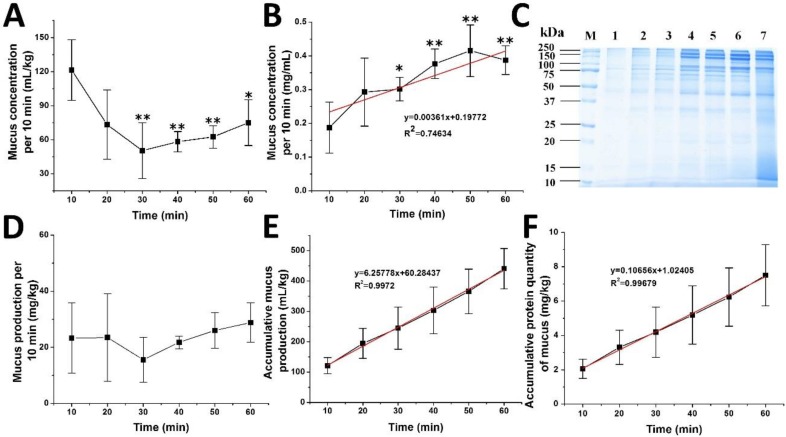
Stress-induced mucus secretion and autolysis of *A. coerulea*. (**A**) Mucus volume of each sample/10 min; (**B**) Protein concentration of each sample/10 min was determined by the Bradford method; (**C**) SDS-PAGE of collected mucus and tissue homogenate of *A. coerulea*. M: protein molecular size marker; Lanes 1–6: mucus samples of *A. coerulea* collected in 60 min; Lane 7: jellyfish tissue homogenate; (**D**) Protein quantity (mg/kg)/10 min; (**E**) Accumulative volume of mucus (mL/kg)/60 min; (**F**) Accumulative protein quantity of mucus (mg/kg)/60 min. Mean ± SD (*n* = 4) is shown. * *p* < 0.05 and ** *p* < 0.01 indicate a significance difference as compared to the control.

External stress was performed by removing *A. coerulea* from the environmental seawater and, as expected, the sticky liquid samples were largely secreted [13] and collected every 10 min for a total of 1 h (Figure 1). Two obvious phases in volume collection were displayed, whereby the volume decreases to a minimum at 30 min, followed by a gradual increase within 60 min (Figure 1A). However, protein concentration of each sample is positively associated with the time (Figure 1B), which is further confirmed by sodium dodecyl sulfate–polyacrylamide gel electrophoresis (SDS-PAGE) (Figure 1C). Proteins in mucus are mainly distributed in three concentrated molecular weight ranges—100–250 kDa, 50–100 kDa and 37–50 kDa—while proteins in tissue are more dispersed. A gentle trough of the curve for protein quantity (mg/kg) of each sample is shown at 30 min (Figure 1D). Meanwhile, obvious crevices in the umbrella part indicate that jellyfish autolysis starts at or even earlier than 30 min. Therefore, the decreases of mucus volume (Figure 1A) and protein amount (Figure 1D) in the first 30 min imply an adaption to the stress while the increase of mucus volume (Figure 1A) and protein amount (Figure 1D) in the latter 30 min is probably due to jellyfish autolysis. Interestingly, straight line correlations (R^2^ > 0.99) for both mucus volume (mL/kg, Figure 1E) and protein quantity (mg/kg, Figure 1F) with time indicate a continuous release of proteins through two different mechanisms—i.e., stress-induced mucus secretion followed by jellyfish autolysis—without clear boundaries. The 20 min sample is less influenced by both residual seawater and jellyfish autolysis, and is therefore the sample selected for proteomics and metabolomics.

### 2.2. Proteomic Comparison of Secreted Mucus and Tissue Homogenate

**Figure 2:**
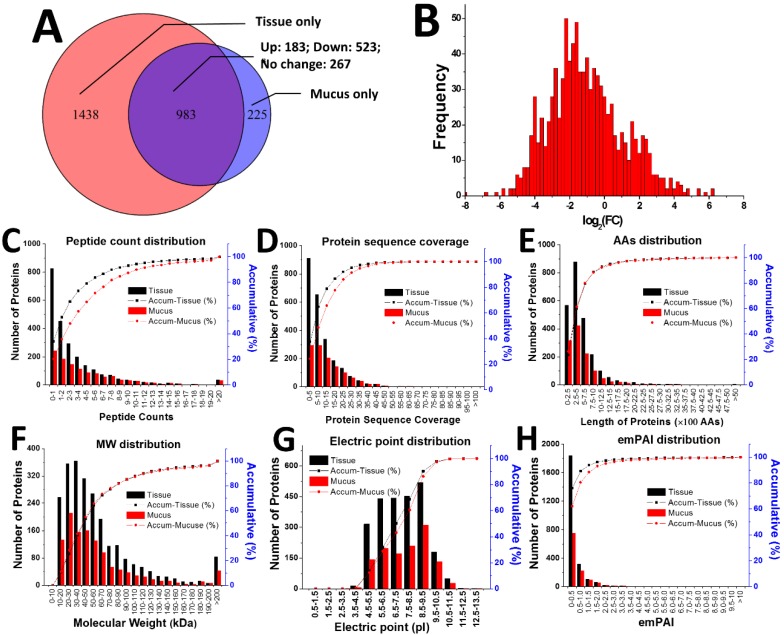
Proteomic comparison of secreted mucus and tissue homogenate. (**A**) Venn diagram of protein composition in mucus and tissue. There were 2421 proteins identified in tissue and 1208 identified in mucus. 1438 and 225 proteins were only found in tissue and mucus, respectively. Of these 983 overlapping proteins in both groups, 183 were found at elevated levels in mucus, while 523 were at lower levels and 267 were at consistent levels when compared to those in tissue. (**B**) Histogram of quantitative ratio of the overlapped proteins between the two groups. The log_2_(FC) (fold change) value from mucus (numerator) vs. tissue (denominator) is distributed mainly between −6 to +6, with a peak located at around −2 instead of 0. Six indexes, including peptide count distribution (**C**), protein sequence coverage (**D**), AA distribution (**E**), MW distribution (**F**), electric point distribution (**G**), and emPAI distribution (**H**), are compared for tissue and mucus.

All proteomics raw MS data were aligned to obtain peptide sequence information and matched to proteins from our previously constructed transcriptomic database for *A. coerulea*. A total of 2729 proteins from 10,560 peptides were identified by LC-MS/MS, where 2421 proteins from 8866 peptides were matched in tissue and 1208 proteins from 4148 peptides were matched in mucus. A Venn diagram shows that 1438 and 225 proteins are separately located in tissue and mucus, respectively. Proteins numbering 183, 523, and 267 are elevated, lowered or unchanged, respectively, among the 983 overlapped proteins in mucus when compared to those in tissue (Figure 2A). This profile is further supported by the quantitative ratio histogram of the overlapped proteins between the two groups where the log_2_(FC) (fold change) value from mucus (numerator) vs. tissue (denominator) values were distributed from −6 to +6, with a peak located at around −2, rather than 0 (Figure 2B). Although protein number of secreted mucus is far less than that of tissue homogenate, two proteomic indexes including amino acid (AA) (Figure 2E) and molecular weight (MW) distributions (Figure 2F) are the same for the two groups. The distribution curves of four other indexes, including peptide count (Figure 2C), protein sequence coverage (Figure 2D), electric point (Figure 2G) and exponentially modified protein abundance index (emPAI) (Figure 2H), are slightly shifted to the right in mucus when compared to those in tissue. These proteomic analysis results indicate that proteins identified in secreted mucus were successfully separated to a high level of purity and that they were independent of those identified in tissue homogenate.

### 2.3. Gene Ontology Analysis

**Figure 3:**
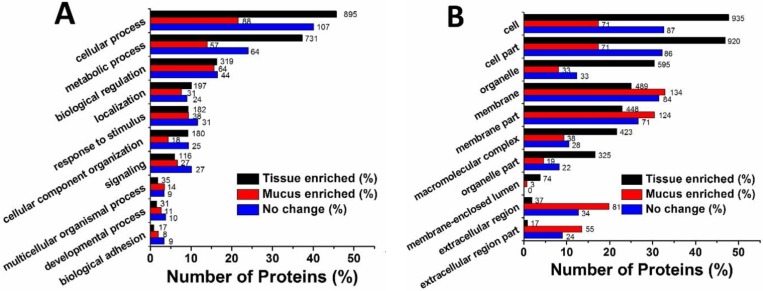

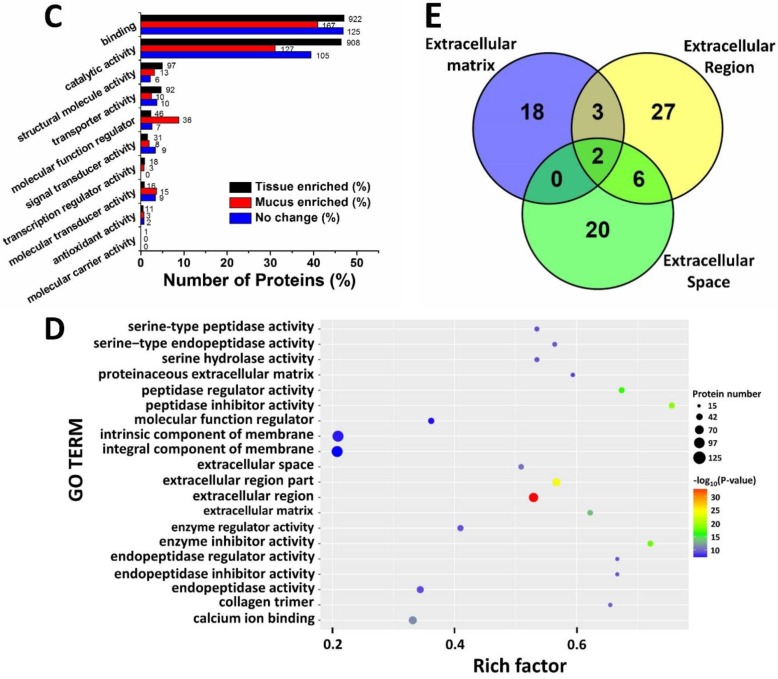
Comparative Gene Ontology (GO) analysis of identified proteins in tissue and mucus of *A. coerulea*. Three groups, namely ‘tissue-enriched proteins’, ‘mucus-enriched proteins’, and ‘proteins with no change’ are displayed. The tissue-enriched proteins or mucus-enriched proteins represent proteins exclusively and highly expressed in tissue homogenate (FC < 0.5) or secreted mucus (FC > 2). The group ‘proteins with no change’ implies that the proteins expressed in both mucus and tissue show no obvious difference (0.5 < FC < 2). (**A**) Biological process (BP). The horizontal axis is the ratio of proteins in the total identified proteins, whereas the vertical axis provides description of the matched GO terms. The protein numbers are labeled on the right side of each transverse column. (**B**) Cellular component (CC). (**C**) Molecular function (MF). (**D**) Diagram of GO enrichment in mucus-enriched proteins. The horizontal axis indicates the rich factor, i.e., the proportion of the number of differentially expressed proteins vs. the total number of proteins in the same GO term. The vertical ordinates represent the matched GO terms. The bubble shows the number of proteins matched in each GO term. The color represents −log_10_(*p* value): Logarithmic conversion of Fisher exact test *p* value. (**E**) Venn diagram of the extracellular proteins in mucus-enriched proteins. Three subclasses ‘extracellular matrix’, ‘extracellular region’ and ‘extracellular space’ are colored by blue, yellow and green, respectively.

The Gene Ontology (GO) project provides an ontology of defined terms describing the characteristics of genes and their products in any organism [24]. It covers three domains: biological process (BP), cellular component (CC), and molecular function (MF). We categorized all identified proteins according to the levels of protein expression in secreted mucus and tissue homogenate. The distributing tendencies of tissue-enriched proteins (FC < 0.5), mucus-enriched proteins (FC > 2) and proteins with no change (0.5 < FC < 2) are similar, although obvious differences are seen in specific terms. Since the quantity of proteins in mucus is much less than that in tissue, we used percentage of the total identified proteins in each group as the horizontal axis, whereas the exact amount of proteins is labeled on the right side of the transverse column (Figure 3A–C).

Among the top 10 terms of BP, the ratios of proteins fall from near 50% in ‘cellular process’ to less than 5% in ‘biological adhesion’ in tissue-enriched proteins. The top three terms, ‘cellular process’, ‘metabolic process’ and ‘biological regulation’ in mucus-enriched proteins have much lower ratios than those in tissue-enriched proteins (Figure 3A). An intermediate distribution is seen in the proteins with no change. The largest difference between the two groups comes from the CC subcategories. Tissue-enriched proteins are mainly distributed in three intracellular locations: ‘cell’, ‘cell part’ and ‘organelle’, then followed by the membrane-related terms ‘membrane’, ‘membrane part’ and ‘macromolecular complex’. Comparatively, the most abundant locations in mucus-enriched proteins are ‘membrane’ and ‘membrane part’. The ratios of protein levels in the intracellular locations ‘cell’, ‘cell part’ and ‘organelle’ are much lower than those in tissue-enriched proteins, while the extracellular terms ‘extracellular region’ and ‘extracellular part’ show elevated percentages of proteins and larger ratios in mucus-enriched proteins when compared to those in tissue-enriched proteins (Figure 3B). The distribution profiles of MF subcategories are similar across all three groups. Interestingly, the ratios of the top two terms, ‘binding’ and ‘catalytic activity’, are close to 50%, which is significantly higher than that of other terms, with ratios of less than 10%. Although the number of ‘molecular function regulator’ proteins is similar between ‘mucus-enriched’ and ‘tissue-enriched’ samples, the ratio of ‘molecular function regulator’ proteins in mucus-enriched proteins is much higher than those in the other two groups, which may potentially be used as the molecular indicators of jellyfish stress (Figure 3C).

We turned our attention to the mucus-enriched proteins, of which the GO enrichment diagram (Figure 3D) is built with the parameters: protein number > 15, rich factor 0.2–0.8 and −log_10_(*p* value) > 7. The most significant feature is that the ‘extracellular region’ shows the highest −log_10_(*p* value) (bright red), although its ‘rich factor’ and ‘protein quality’ are not the largest. Moreover, a Venn diagram was constructed to further divide the extracellular proteins in mucus-enriched proteins into three subclasses—‘extracellular region’, ‘extracellular matrix’ and ‘extracellular space’—with 23, 32 and 28 proteins, respectively, in each subclass. The ‘extracellular region’ and ‘extracellular matrix’, ‘extracellular region and extracellular space’, ‘extracellular space and extracellular matrix’ share five, eight and two proteins, respectively. Only two proteins overlap across all three subgroups (Figure 3E).

### 2.4. KEGG Pathway Analysis

**Figure 4:**
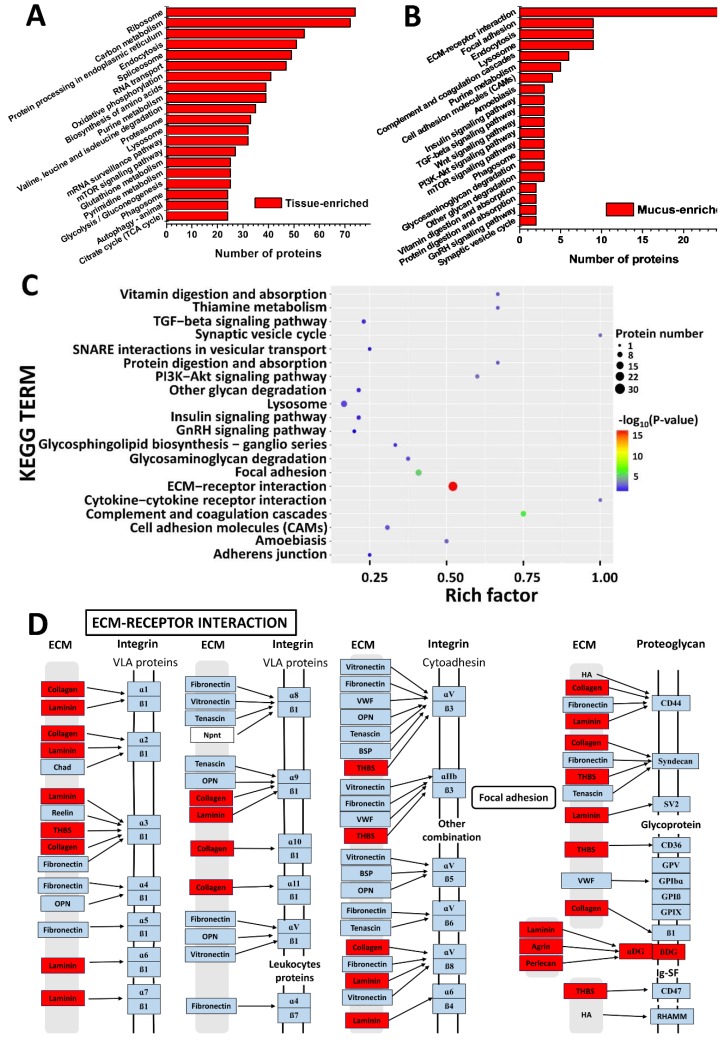
Kyoto Encyclopedia of Genes and Genomes (KEGG) pathway annotation of identified proteins in tissue and mucus of *A. coerulea*. (**A**) KEGG pathway annotation of tissue-enriched proteins. The horizontal axis is the number of proteins, whereas the vertical ordinates are the terms of the KEGG pathways. (**B**) KEGG pathway annotation of mucus-enriched proteins. (**C**) KEGG pathway enrichment of mucus-enriched proteins. The horizontal axis indicates rich factor and vertical ordinates are the terms of the KEGG pathways. Rich factor is the proportion of the number of differentially expressed proteins vs. the total number of proteins in the same KEGG pathway. The bubble shows the number of proteins matched in the KEGG pathway. The color represents −log_10_(*p* value): Logarithmic conversion of Fisher exact test *p* value. (**D**) The ‘ECM-receptor interaction’ pathway matched from the KEGG PATHWAY database where the elevated proteins from mucus-enriched proteins are colored by red.

KEGG (Kyoto Encyclopedia of Genes and Genomes) is a common bioinformatics tool and was utilized in this study to provide pathway mapping of identified proteins. The top 20 matched pathways among the 226 successfully mapped pathways are mainly associated with the intracellular synthesis of metabolites, as well as intracellular functions (Figure 4A) in tissue-enriched proteins. The number of proteins gradually falls from 74 in the ‘Ribosome’ and 72 in ‘Carbon metabolism’ to only one protein in each pathway. By comparison, only 81 pathways were matched in mucus-enriched proteins. There are 25 proteins matched to the ‘ECM (extracellular matrix)—receptor interaction’ pathway, significantly more than in other pathways (Figure 4B). The largest spot (shown in red) has a rich factor of 0.52 and represents an ‘ECM-receptor interaction’. This is shown in the KEGG enrichment diagram for protein-enriched mucus (Figure 4C). Three subclasses, namely collagen, laminin and thrombospondin (THBS) are further divided from the 25 matched proteins identified in the ‘ECM-receptor interaction’ that are known to function in the matrix-receptor interactions (Figure 4D).

### 2.5. Metabolomics

**Figure 5:**
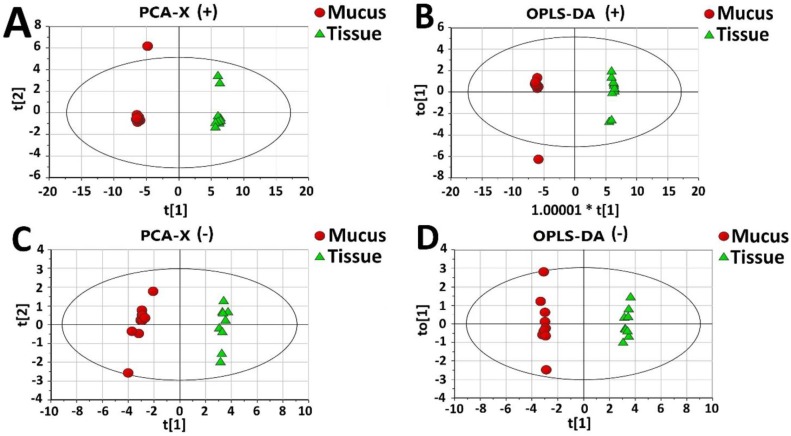

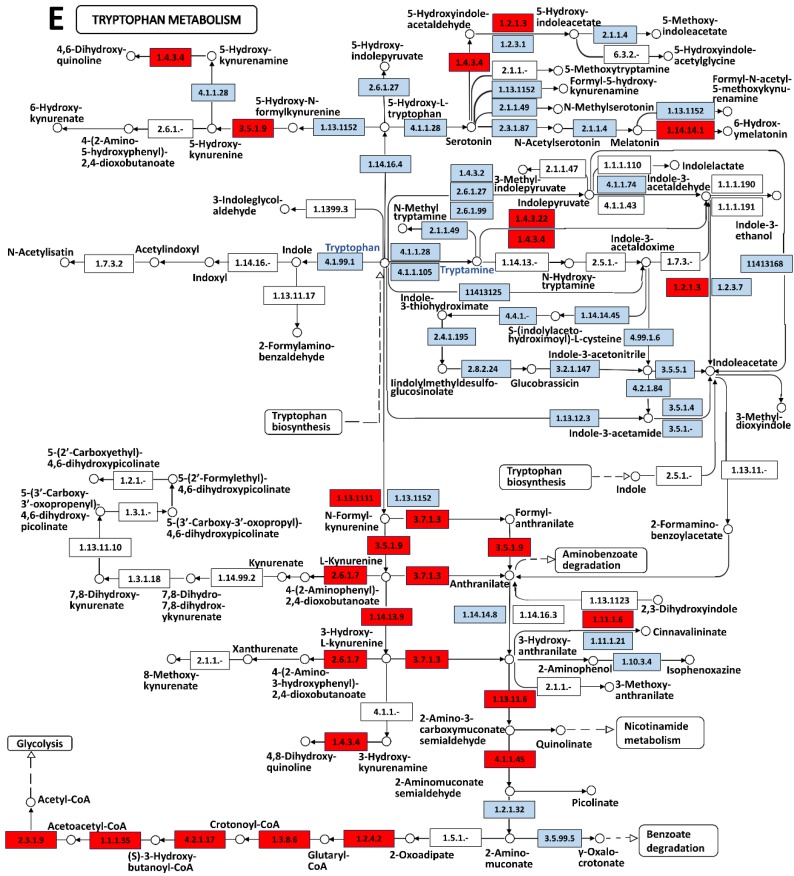
Plots of multivariate statistical analysis of all experimental groups in electrospray ionization (ESI) positive and negative-ion MS detection modes. The difference in substances between the two groups was screened by a variable importance plot (VIP). (**A**) Principal component analysis-X variogram (PCA-X) (+) and (**B**) Orthogonal partial least-squares-discriminant analysis (OPLS-DA) (+) scores plot of the mucus and tissue groups in the ESI positive-ion mode. Asterisk (*) indicates multiplication sign. (**C**) PCA-X (−) and (**D**) OPLS-DA (−) scores plot of the mucus and tissue groups in the ESI negative ion mode. The red plots represent the data from the secreted mucus, whereas the green plots represent data from the tissue homogenate. (**E**) The ‘Tryptophan metabolism’ pathway from KEGG PATHWAY database where the mRNAs from the transcriptomics of jellyfish tissue are colored by red, while the light blue and white boxes represent the background genes annotated or not in the KEGG PATHWAY database. Meanwhile, the metabolites ‘Tryptophan’ and ‘Trytamine’ are highlighted by blue.

Besides the protein or peptide components, metabolites in mucus were also determined by UPLC-QTOF MS/MS and identified from common metabolite databases by comparing the molecular weights. Principal component analysis (PCA) provides a summary of all the observations, revealing significant differences between mucus and tissue in both positive-ion (Figure 5A) and negative-ion (Figure 5C) detection modes. Similar results were obtained by orthogonal partial least-squares-discriminant analysis (OPLS-DA) and was subsequently used to determine the most significant metabolites in mucus and tissue in both positive-ion (Figure 5B) and negative-ion (Figure 5D) MS detection modes using the variable important plot (VIP) value (>1). The R2X, R2Y, and Q2Y values in the positive ion mode were 0.884, 0.999 and 0.997, respectively, whereas their values in negative ion mode were 0.712, 0.998 and 0.993, respectively, indicating a good predictive power and goodness-of-fit of OPLS-DA plots.

A total of 16 discriminating metabolites with three subgroups were obtained (Table 1) according to FC values of mucus vs. tissue. The FCs of nine metabolites including l-Glutamate, Succinylacetone, Linoleyl linolenate, Uridine, l-Proline, Inosine, Hypoxanthine, l-Valine and Guanosine are smaller than 1, representing a lower concentration in mucus than that in tissue. Five metabolites including 4-Hydroxy-l-proline, Citrulline, l-Leucine, 3-(Phosphoacylase mido)-l-alanine and l-Threonine have FC values ≈ 1, indicating similar concentrations between the two groups. The metabolite Tryptamine, a derivative of Tryptophan, is a potential neurotransmitter or neurotransmodulator, and displayed the largest FC value of 7.8. This value was significantly higher than all other values and indicates elevated enrichment in mucus over that of all other metabolites. This feature is of particular interest to us. A further KEGG scanning of Tryptamine shows that there are 16 proteins identified from proteomics and 26 mRNAs from transcriptomics (Figure 5E) in tissue homogenate. Only 1 ‘mucus-enriched’ protein identified was matched to the downstream region of the ‘Tryptophan metabolism’ pathway. This indicates a strong Tryptophan/Tryptamine metabolic system in the cytosol, but not in mucus, allowing for Tryptamine synthesis or accumulation.

### 2.6. Self-Protective Proteins

When danger or other stresses arise, a large secretion of mucus occurs as an emergent self-protective reaction. According to our GO and KEGG data, some of the mucus-enriched proteins identified relate to immunization or prevention mechanisms against harmful or invading organisms. Therefore, we have identified these as self-protective proteins of jellyfish mucus. On the whole, 11 metalloproteinases, 4 serine proteinase inhibitors, 3 superoxide dismutases (SODs) and 3 complements were successfully scanned (Table 2, Supplementary Table S1).

#### 2.6.1. Metalloproteinases

**Figure 6:**
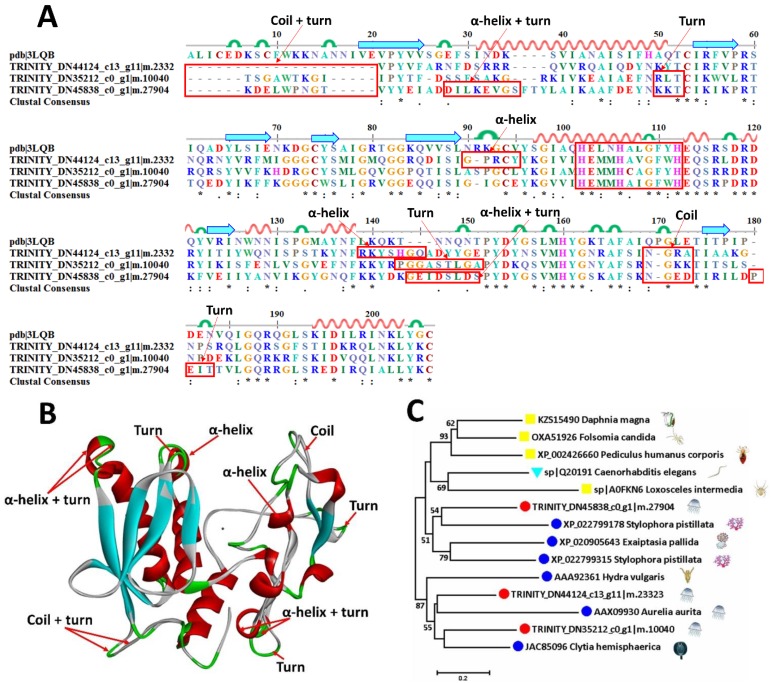
Sequence alignment, 3D modeling and phylogenetic analysis of putative zinc metalloproteinases from *A. coerulea*. (**A**) Three putative sequences TRINITY_DN45838_c0_g1|m.27904, TRINITY_DN44124_c13_g11|m.23323, and TRINITY_DN35212_c0_g1|m.10040 in mucus-enriched proteins are aligned with a model metalloproteinase (pdb ID: 3LQB). At the bottom of columns, asterisks (*) show conserved positions, colons (:) show conserved substitutions and points (.) show non-conserved substitutions. Grey line, green bend, blue banded arrowhead and red solenoid represent coil, turn, sheet and helix, respectively. Different fragments are framed by red lines. (**B**) 3D modeling was simulated using the template metalloproteinase (pdb ID: 3LQB) by SWISS-MODEL and viewed by Discovery Studio 4.5. The colors grey, green, blue and red represent coils, turns, sheets and helices, respectively. Different fragments are indicated by red arrows. (**C**) Phylogenetic tree constructed using three putative zinc metalloproteinases and 11 other sequences from different species using MEGA 7 with the Neighbor-Joining method.

Metalloproteinases are enzymes characterized by a catalytic zinc ion in its active site [25]. They have been described as the toxic components responsible for the induction of tissue damage, necrosis and hemorrhage [26,27] in various venoms [28,29,30]. Eleven metalloproteases with four subgroups—zinc metalloproteinase, matrix metalloproteinase (MMP) [31], A disintegrin and metalloproteinase domain-containing protein (ADAM) [32,33], and A disintegrin and metalloproteinase with thrombospondin motifs (ADMTMS) [34,35]—are predicted with their similarity 30.6–42.6% in mucus-enriched proteins of *A. coerulea* (Table 2). All three predicted zinc metalloproteinases display a relatively conserved astacin-like subfamily segment with a HEXXH or even more strict HEXXHXXGXXH zinc-binding site/active site [36,37]. A model metalloproteinase (pdb ID: 3LQB) is utilized to do the sequence alignment (Figure 6A) and 3D modeling (Figure 6B). With the exception of the highly conserved HEXXHXXGXXH motif, four parallel β-sheets and three α-helixes are also structurally similar. The differences include an initial coil and turn in all three sequences, an extra α-helix and a turn in ^28^DILKEVGS^35^ of TRINITY_DN45838_c0_g1|m.27904, an α-helix in ^90^GPRCY^95^, a changeable area with an α-helix in ^139^RKYSHGQ^145^ of TRINITY_DN44124_c13_g11|m.23323, a turn in ^143^PGGASTLGA^152^ of TRINITY_DN35212_c0_g1|m.10040 and an α-helix and turn in TRINITY_DN45838_c0_g1|m.27904, an extra coil between 169 and 173 of all three predicted sequences, and a turn in ^169^PEIT^183^ of TRINITY_DN45838_c0_g1|m.27904. Interestingly, two sequences—TRINITY_DN45838_c0_g1|m.27904 and TRINITY_DN44124_c13_g11|m.23323—aligned to the same metalloproteinase, nas-13, display a large molecular evolutional distance, and are close to XP 022799178.1 from the coral *Stylophora pistillata* and AAX09930.1 from the same jellyfish *A. coerulea*, respectively. Meanwhile, the remaining sequence, TRINITY_DN35212_c0_g1|m.10040, is evolutionally close to a sequence JAC85096 from *Clytia hemisphaerica* (Figure 6C).

#### 2.6.2. Serine Protease Inhibitors

**Figure 7:**
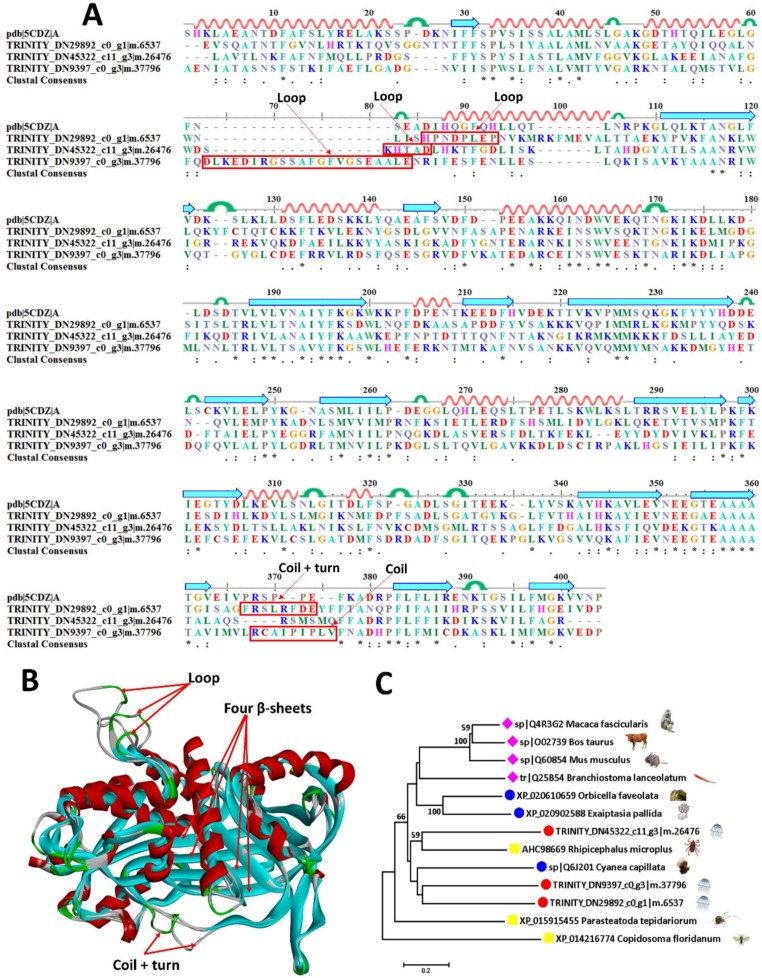
Sequence alignment, 3D modeling and phylogenetic analysis of the putative serpins from *A. coerulea*. (**A**) Three putative sequences TRINITY_DN45322_c11_g3|m.26476, TRINITY_DN9397_c0_g3|m.37796, and TRINITY_DN29892_c0_g1|m.6537 in mucus-enriched proteins are aligned with a model serpin (pdb ID: 5CDZ). At the bottom of columns, asterisks (*) show conserved positions, colons (:) show conserved substitutions and points (.) show non-conserved substitutions. Grey line, green bend, blue banded arrowhead and red solenoid represent coil, turn, sheet and helix, respectively. Different fragments are framed by red lines. (**B**) 3D modeling was simulated using the template serpin (pdb ID: 5CDZ) by SWISS-MODEL and viewed by Discovery Studio 4.5. The colors grey, green, blue and red represent coils, turns, sheets and helices, respectively. Different fragments are indicated by red arrows. (**C**) Phylogenetic tree constructed by three putative serpins and 10 other sequences from different species using MEGA 7 with the Neighbor-Joining method.

Serine protease inhibitors or serpins [38] are the largest and most diverse superfamily of protease inhibitors [39], with inhibitory or non-inhibitory functions in blood coagulation, fibrinolysis, host defense, and impairment of motility of human glioblastoma cells [29,40,41]. They have widely been found in the venoms of many poisonous animals such as sea anemones, snakes, scorpions, spiders, anurans and hymenopterans [42]. Four sequences have been successfully scanned with their similarities identified at 36.6–56.4% to mucus-enriched proteins of *A. coerulea* (Table 2). Similar to zinc metalloproteinases, serpins are more similar in spatial structure than amino acid sequences simulated with a model serpin (pdb ID: 5CDZ) (Figure 7A,B). Two clusters of β-sheets are distributed in the central and tail areas, whereas 11 α-helixes are scattered in the periphery of proteins. The main structural variations contain an extended loop in ^86^HPNDPLEP^93^ of TRINITY_DN29892_c0_g1|m.6537, ^82^KHTAD^86^ of TRINITY_DN45322_c11_g3|m.26476 and ^63^DLKEDIRGSSAFGFVGSEAALE^84^ of TRINITY_DN9397_c0_g3|m.37796, an extended coil and turn in ^367^FPSLRFDE^374^ of TRINITY_DN29892_c0_g1|m.6537, and an extended coil in ^368^RCAIPIPLV^376^ of TRINITY_DN9397_c0_g3|m.37796. Phylogenetic analysis shows that TRINITY_DN45322_c11_g3|m.26476 and TRINITY_DN29892_c0_g1|m.6537 are close to AHC98669 in *Rhipicephalus microplus* while TRINITY_DN9397_c0_g3|m.37796 is near XP_015915455 in *Parasteatoda tepidariorum* (Figure 7C).

#### 2.6.3. Superoxide Dismutase

**Figure 8:**
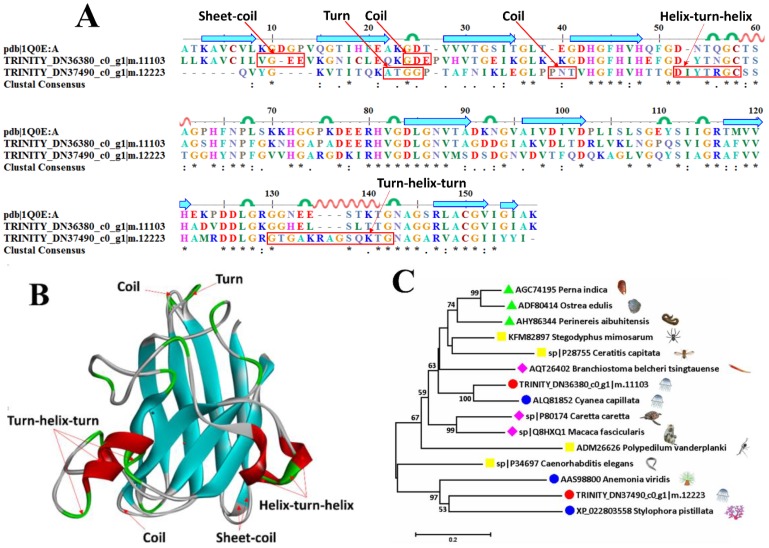
Sequence alignment, 3D modeling and phylogenetic analysis of the putative Cu/Zn superoxide dismutases (SODs) from *A. coerulea*. (**A**) Two putative sequences, TRINITY_DN36380_c0_g1|m.11103 and TRINITY_DN37490_c0_g1|m.12223, in mucus-enriched proteins are aligned with a model SOD (pdb ID: 1Q0E). At the bottom of columns, asterisks (*) show conserved positions, colons (:) show conserved substitutions and points (.) show non-conserved substitutions. Grey lines, green bends, blue-banded arrowheads and red solenoids represent coils, turns, sheets and helices, respectively. Different sections are framed by red lines. (**B**) 3D modeling was simulated using the template SOD (pdb ID: 1Q0E) by SWISS-MODEL and viewed by Discovery Studio 4.5. The colors grey, green, blue and red represent coils, turns, sheets and helices, respectively. Different sections are indicated by red arrows. (**C**) Phylogenetic tree constructed by two putative SODs in mucus-enriched proteins and 13 representative sequences from different species by MEGA 7 with the Neighbor-Joining method.

The serum superoxide dismutases (SODs) could effectively eliminate reactive oxygen species (ROS) and maintain the redox balance [43], thereby playing a key role in protection against oxidative tissue injury in both prokaryotes and eukaryotes [44,45,46,47,48]. Three sequences were successfully scanned with their similarities identified at 51.8–75.3% in mucus-enriched proteins of *A. coerulea* (Table 2). A SOD (pdb ID: 1Q0E) was selected as the model sequence (Figure 8A) and structural template (Figure 8B) of the two putative Cu/Zn-SODs. The core is constituted of two groups of parallel β-sheets that are surrounded by scattered helixes, turns and coils. The discrepancies include an extra helix-turn-helix in ^52^DIYTRGC^58^ and an extended 130GTGAKRAGSQKTG^142^ of TRINITY_DN37490_c0_g1|m.12223. Phylogenetic analysis shows that TRINITY_DN36380_c0_g1|m.11103 is close to ALQ81852 in *Cyanea capillata* while TRINITY_DN37490_c0_g1|m.12223 is near XP_022803558 in *Stylophora pistillata* (Figure 8C).

## 3. Discussion

### 3.1. Jellyfish Mucus Is a Rich Library for the Discovery of Novel Bioactive Compounds

Mucus provides a unique and multi-functional hydrogel interface between the epithelial cells and their external environment [49]. In most phyla of aquatic and terrestrial metazoans, it has exceptional properties, including elasticity, changeable rheology and an ability to self-repair, and therefore is an ideal medium for trapping and immobilizing pathogens [50]. Moreover, mucus is a rich library of bioactive components functioning against invasion or microorganisms in innate immunity. Sea star integument produces mucus with antioxidant proteins such as peroxiredoxin, catalase and SODs, and anti-microbial proteins such as lysozyme, melanotransferrin and ribosomal proteins [18]. Fish skin mucus serves as the first line of defense against pathogens and external stressors with antioxidant proteins, lectin, calmodulin, histone proteins, Cystatin-B, Apolipoprotein A1, and heat shock proteins [15]. In the human body, the protective mucus widely covers the epithelial cells on the surface of various tissues and organs such as respiratory organs, stomach, intestine, genitourinary organs, etc. [51,52,53]. Nasal mucus plays crucial roles in preventing microbial infections and protecting the lower airways from the unhealthy conditions of ambient air through fibrinogen, plasminogen, and complement factor C3 [54], whereas self-protective proteins from cervical mucus include serpins, phosphorylated proteins and heat-shock proteins [55].

In recent years, the large-scale outbreak of jellyfish blooms and dramatic increase of patients by jellyfish envenomation has drawn great attention in both fishery and medicine [56]. Immediate pain, redness and swelling occurs on local skin after being injured by contacting the tentacles of jellyfish, while serious systemic symptoms include multiple organ failures or even death [57]. The venom that is released from nematocyst of jellyfish tentacle possesses a large amount of toxins and other active proteins leading to these various manifestations, and is therefore naturally considered to be a rich library of bioactive compounds. Using the integrated methods of traditional liquid chromatography, amino acid sequencing and cDNA library alignment, the hemolytic proteins were firstly purified and identified from two box jellyfish, *Carybdea rastoni* [58] and *Carybdea alata* [59], in 2000; subsequently, more than ten hemolytic proteins have successfully been identified as a novel cytotoxic protein family [60,61,62]. By the combination of transcriptomics and proteomics, more than 170 potential toxin proteins, including metalloproteinases, an alpha-macroglobulin domain containing protein, two CRISP proteins, a turripeptide-like protease inhibitor, and particularly, nine novel members of a taxonomically restricted family of cnidarian pore-forming toxins, were successfully identified from the jellyfish *Chironex fleckeri* on the basis of homology to known toxins in public sequence databases [63]. Similarly, 174 potential toxic proteins with 27 homologs to the toxins from venomous animals, including phospholipase A2, zinc metalloproteinase-disintegrin agkistin, serine protease inhibitor, plancitoxin-1, alpha-latrocrustotoxin-Lt1a, etc. were scanned in the jellyfish *Cyanea nozakii* [64].

When an envenomation happens, the skin firstly contacts the jellyfish mucus; this is also an important poisoning source described by fishers indicating that mucus also contains a large number of active components potentially deriving from nematocyst venoms. Because mucus covers the surface of tentacles, including nematocysts, any release of nematocyst venom has a certain portion of debris, thereby displaying a conventional material communication with mucus. Meanwhile, considering the long evolutionary history of jellyfish and the key role of mucus in innate immunity of other aquatic organisms, there should also be a large number of bioactive compounds to function as adhesion and in defense. In this study, we explored the stress-induced mucus secretion and its composition by a combination of proteomics and metabolites using a low-toxicity jellyfish, *A. coerulea*. We successfully scanned 1208 and 2421 proteins in mucus and tissue, respectively. Among them, 225 proteins are exclusively identified in mucus while 183 proteins are up-regulated when compared to that of the tissue. As expected, the mucus-enriched proteins possess dozens of functions as indicated by GO and KEGG analyses. Moreover, 21 self-protective proteins, such as metalloproteinases, serine proteinase inhibitors, SODs and complements were successfully scanned against potential external invasion.

### 3.2. Tryptamine Release Indicates an Elevated Stress of Jellyfish When Stimulated

Metabolomics is a technique for studying metabolic networks in biological systems by examining the metabolite profiles and their dynamic changes before and after stimulation or disturbance [65,66]. The number of metabolites is much less than those of genes and proteins, and small changes in gene and protein expression can be amplified at the metabolite levels [67]. In this study, we have found three groups of metabolites with lower, equal and higher expressions in mucus when compared to those in tissue by metabolomics. Because metabolites in mucus have the tendency to disperse into the surrounding seawater [68,69] and all are feasibly synthesized and secreted from jellyfish tissue, it is reasonable that metabolites with lower concentrations (l-Glutamate, Succinylacetone, Linoleyl linolenate, Uridine, l-Proline, Inosine, Hypoxanthine, l-Valine and Guanosine) in mucus are in normal conditions while those with equal concentrations (4-Hydroxy-l-proline, Citrulline, l-Leucine, 3-(Phosphoacylase mido)-l-alanine and l-Threonine) should already have been well enriched with the balance between release and diffusion.

Of particular interest, the metabolite Tryptamine displays the maximal FC value of 7.8, which is far higher and indicates much better enrichment than that of other metabolites. Tryptamine is a group of monoamine alkaloids including serotonin (5-hydroxytryptamine, 5-HT) and melatonin, as well as other compounds known for their neurotransmitter properties [70]. It derives from the amino acid-trytophan by tryptophan decarboxylase (EC 4.1.1.105 and EC 4.1.1.28), which is also named aromatic l-amino acid decarboxylase (AADC or AAAD), DOPA decarboxylase (DDC) and 5-hydroxytryptophan decarboxylase, and which plays an important role in the dopaminergic system participating in the uptake and decarboxylation of amine precursors in the peripheral tissues [71,72]. The typical tryptamine, serotonin, is one of the most important and widely studied hormones in humans and other vertebrates involved in regulation and modulation of multiple processes within the central nervous system and behavior [73]. It also plays an important role in gastrointestinal motility, vascular tone and platelet function, and has been related to various pathophysiological processes [74]. However, less research on tryptamine is reported in marine invertebrates. It is reported that cells with serotonin are concentrated at the anterior pole of hydrozoan planulae [75]. Brain serotonin levels in crayfish are reported to greatly increase when exposed to pressure [76]. Because jellyfish have a well-developed peripheral nervous system, and it is reported that tryptamine can be accumulated and released in large quantities under stress in marine invertebrates [76,77], we hypothesize that the increases of tryptamine release and mucus secretion indicate an elevated stressful response when stimulated by taking the jellyfish out of seawater. This will provide a new mechanism for jellyfish to secrete mucus although further validation is needed.

### 3.3. Self-Protective Proteins Play a Key Role in Innate Immunity of Jellyfish

In mucus-enriched proteins, we have successfully scanned dozens of self-protective proteins including 11 metalloproteinases, four serine protease inhibitors, three SODs, and three complements. According to the activity of metalloproteinase that degrades extracellular matrix proteins and bioactive molecules on the surface of the jellyfish *A. coerulea*, three functions are proposed as an important self-protective factor. The first function is to process the extracellular matrix such as collagen, laminin and THBS to form the main component of the mucus [78]. The second self-protective effect is to directly digest or degrade the invasive toxic component or microorganism, which can further induce the third function, transferring the external stimuli to the body by activating or deactivating the signaling through cutting or digesting the extracellular part of membrane proteins such as Her2 receptors. On the contrary, serine protease inhibitors form stable complexes with their target enzymes to control the activity of serine proteases [79], thereby playing an important role in innate immunity and environmental stability in the body [80]. On the surface of jellyfish, serine protease inhibitors are able to prevent the over-digestion of functional proteins through inhibiting the serine proteases from both jellyfish themselves and the exogenous invading pathogens. As the natural antioxidant function, SODs in jellyfish mucus should catalyze the production of O_2_ and H_2_O_2_ from superoxide (O_2_^−^), which results in less harmful reactants and protects cellular components from being oxidized by ROSs [81,82] that are feasibly from jellyfish tissue, invading microorganisms or environmental conditions such as water pollution, eutrophication, anoxia and radiation. In addition to the above, we also listed three complements. The complement system is a highly complicated defense system in the innate immunity of invertebrates, which directly participates in the lysis of pathogen cell [83]. Most studies on cnidarian complement have focused on C3 [83,84], of which the activation plays a central role and is required for all three pathways of complement activation [85]. By comparison, the complement factor B is a component of the alternative pathway of complement activation to form the pore complexes and lyse the invading micrograms.

## 4. Materials and Methods

### 4.1. A. coerulea Samples

*A. coerulea* were collected alive from an artificial aquafarm in Shanghai, China in May 2017. Jellyfish were transported in a 3-L plastic bag fully filled with seawater, to prevent damage from sloshing. In the laboratory all individuals were maintained in buckets of seawater at 18–22 °C.

### 4.2. Transcriptome Sequencing and Its Annotation

Total RNA of jellyfish *A. coerulea* was extracted with TRIzol (Life Technologies) following the manufacturer’s procedure. RNA purity, concentration and integrity were evaluated using NanoDrop 2000 UV–Vis spectrophotometer (Thermo Fisher Scientific, Waltham, MA, USA), Agilent 2100 Bioanalyzer (Agilent Technologies, Santa Clara, CA, USA) and RNA 6000 Nano LabChip® Kit (Agilent Technologies, Santa Clara, CA, USA). The mRNA was enriched by polyT oligo-conjugated magnetic beads, then fragmentation buf Santa Clara, America fer was added to break the mRNA into short fragments. A single strand of cDNA was synthesized by using random hexamers as template. The double-stranded cDNA was synthesized by adding buffer, dNTPs, DNA polymerase I and RNase H. The double-stranded cDNA was purified by AMPure XP beads (Beckman, Atlanta, GA, USA). The purified double-stranded cDNA was repaired at the end, A-tail was added and sequencing junction was connected, and AMPure XP beads were used to select the fragment size. Finally, PCR was amplified and purified by AMPure XP beads, and the final cDNA library was obtained. Illumina HiSeq sequencing was carried out after qualification.

The raw data of sequencing were evaluated by FastQC (version: 0.11.2) and filtered by Trimmomatic (version: 0.36) to remove the joints as well as the sequences with low quality. Then the clean data were de novo assembled into transcripts by Trinity, which were further annotated by blast using the databases NR (National Center for Biotechnology Information non-redundant protein sequences) and Swissprot. According to the annotation results of transcripts NR and Swissprot, GO and KEGG analyses were finally performed according to the annotation of transcripts by NR and Swissprot.

### 4.3. Mucus Collection and Tissue Homogenate Preparation

The jellyfish *A. coerulea* were fasted for 48 h and gently washed thoroughly with sterile filtered artificial seawater before experiments. After measurement of body weight, jellyfish were gently put into a funnel with one layer of medical gauze, which was then sealed with a plastic wrap to avoid liquid evaporation. Jellyfish mucus was collected every 10 min with a 15 mL centrifuge tubes, for a total of 1 h. The debris was removed by centrifugation at 4000 rpm for 10 min and the supernatant was collected and stored at −80 °C for further experiments. Jellyfish tissue homogenate was obtained using the method of ultrasonic extraction. In every working cycle, the working time and resting time were 20 s and 1 min with a power of 200 W. The total working time was 2 min. The supernatant of jellyfish tissue homogenate was collected after centrifugation at 4000 rpm for 10 min at 4 °C, which was then stored at −80 °C for further analysis.

Four replicates of all experiments were performed, using independent batches of *A. coerulea* samples. Protein concentrations of both mucus and tissue homogenate were determined by Bradford’s assay. Bovine Serum Albumin (BSA) was used to construct the standard curve.

### 4.4. SDS-PAGE

Jellyfish mucus and tissue homogenate were assessed by sodium dodecyl sulfate polyacrylamide gel electrophoresis (SDS-PAGE) using 5% stacking gel and 10% resolving gel. Samples were mixed with 5× Loading Buffer (*v*/*v* = 4:1) and then heated at 100 °C for 5 min prior to loading. Eight samples including six mucus (10–60 min), one tissue homogenate and the protein marker with the molecular weight scale 2–250 kDa were electrophoresed simultaneously. The selected voltages were 120 V and 180 V for stacking and resolving gel (PowerPac™ Basic, Bio-Rad, Hercules, CA, USA), respectively. To reduce the staining background as well as to improve the dye sensitivity, the PAGE gel was boiled in deionized water for 3 min and then shaken at 60–70 rev/min for 5 min in a rotary shaker. Subsequently, the PAGE gel was stained with Coomassie Brilliant Blue R-250 staining (Beyotime, Shanghai, China) at 60–70 r/min for 30 min, decolored with the deionized water overnight, and finally scanned for images (Perfection V700 Photo, Epson, Suwa, Japan).

### 4.5. Proteomics

#### 4.5.1. Protein Sample Preparation

The proteins of jellyfish mucus and tissue homogenate were precipitated with four times the volume of trichloroacetic acid (TCA) at 4 °C overnight. The sediment was collected after centrifugation at 15,000× *g* for 15 min at 4 °C, further treated with a pre-cooled acetone solution for 30 min, and re-centrifuged at 15,000× *g* for 15 min at 4 °C. After freeze-drying, the precipitates were dissolved in the lysis buffer (8.0 M Urea, 1× Protease inhibitor, 100 mM Tris-HCl, pH 8.0) overnight. Subsequently, the undissolved debris was removed by centrifugation at 15,000× *g* for 15 min at 4 °C, whereas the supernatant was stored at −80 °C for further use.

After mass quantification of the pre-treated samples, 60 μg mucus or tissue homogenate were mixed with 5 μL 1 M dithiothreitol (DTT) at 37 °C for 1 h, and reacted with 20 μL 1 M indoleacetic acid (IAA) in the dark at room temperature for 1 h. After that, samples were collected by centrifugation in ultrafiltration tubes and sequentially rinsed three times with 100 μL UA solution (8 M urea, 100 mM Tris-HCl, pH 8.0) and 100 μL 50 mM NH_4_HCO_3_, respectively. Finally, the collected samples were digested with Trypsin (protein:trypsin = 50:1) at 37 °C for 12–16 h, and the digested proteins were lyophilized and stored at −80 °C for further use.

#### 4.5.2. LC-MS/MS

Samples were analyzed by liquid chromatography-mass spectrometry/mass spectrometry (LC-MS/MS) using an Orbitrap Fusion Lumos mass spectrometer coupled to an Easy nLC/Ultimate 3000 nano-HPLC chromatography system (Thermo Fisher Scientific, Waltham, MA, USA). In the process of separation, the column was equilibrated with 95% buffer A (0.1% formic acid). The digested proteins were desalted with a C18 pre-column (3 mm, 100 μm × 20 mm, Thermo Scientific, USA). After loading and washing the digested proteins, the separation was performed with an analytical C18 column (1.9 mm, 150 μm × 120 mm, Thermo Scientific, USA) at a flow rate of 600 nL/min for 75 min. The mobile phase was the mixture of the buffer A (0.1% FA, H_2_O) and the buffer B (0.1% formic acid (FA), 100% acetonitrile (ACN)). Elution gradient parameters were setup as follow: 0–14 min, 7–13% buffer B; 14–51 min, 13–23% buffer B; 51–68 min, 23–36% buffer B; 68–69 min, 36–100% buffer B; and 69–75 min, 100% buffer B. Mass spectrometric analyses were carried out by an automated data-dependent Tandem Mass Spectrometry (MS/MS) analysis with full scans (300–1400 *m*/*z*) that was acquired from proteins in the Orbitrap at a mass resolution of 120,000. The positive ion mode and the negative ion mode were separately employed with the spray voltage of the mass spectrometer at 2000 V and 600 V, and the spray temperature of 320 °C for peptides. Normalized collision energy was set to 35% and the stepped collision energy was 5%. Automatic gain control settings for Fourier Transform Mass Spectrometry (FTMS) survey scans were 500,000 and for FT MS/MS scans 5000. Maximum injection time was 50 ms for survey scans and 35 ms for MS/MS scans.

#### 4.5.3. Database Search and Bioinformatics Analysis

All raw data were aligned from the *A. coerulea* transcriptomic database, which was previously built by us. Raw MS files were processed for the peptide analysis using the software Proteome Discoverer 1.4 (ver. 1.4.0.2888; Thermo Fisher Scientific, Waltham, MA, USA). The parameters used for data analysis were: enzyme = trypsin, max missed cleavages = 2, fix modifications = cysteine carbamido methylation, variable modifications = methionine oxidation, *N*-terminal acetylation, peptide mass tolerance = ±15 ppm, and fragment mass tolerance = 20 mmu. The false discovery rate (FDR) < 0.01 was selected for peptide and protein identification. Differential protein screening was performed at a threshold of 2 fold change (FC). FC ≥ 2, FC ≤ 0.5 and 0.5 ˂ FC < 2 representing, up, down and no significant change in the data of protein expression, respectively.

The bioinformatics of Gene Ontology (http://www.geneontology.org, GO) was analyzed on the differentially expressed proteins with a 2 FC according to biological processes, cellular components and molecular functions. GO enrichment was analyzed on the differentially expressed proteins with *p* ≤ 0.05 that was calculated based on a hypergeometric distribution with the default database as the background. The signal pathways were analyzed by the primary public database Kyoto Encyclopedia of Genes and Genomes (KEGG) (http://www.kegg.jp/kegg/pathway.html), and enriched by the tool Pathway Maps. *p*-values were calculated based on a hypergeometric distribution with the default KEGG database as the background. Multiple sequence alignment analysis was performed with BioEdit software (http://www.mbio.ncsu.edu/bioedit/page2.html) under default parameters. RNA sequences of *A. coerulea* were translated into protein through ORFfinder online service (https://www.ncbi.nlm.nih.gov/orffinder). Sequences used for multiple sequence alignment were collected from SwissProt (http://www.uniprot.org/uniprot) or NCBI (https://blast.ncbi.nlm.nih.gov/Blast.cgi) databases, and performed with CLUSTALW program using Bioedit software (version: 7.0.5.3). Finally, phylogenetic trees were constructed using MEGA7 using the Neighbor-Joining method, whereas the 3D modeling was carried out by the combination of the online service SWISS-MODEL (https://swissmodel.expasy.org/) and software Discovery studio 4.5.

### 4.6. Metabolomics

#### 4.6.1. Sample Preparation

Jellyfish mucus and tissue homogenate were precipitated with three times the volume of methanol. The precipitates were removed by 4000 rpm centrifugation for 10 min at 4 °C after shaking of the mixture for 1 min. The clear supernatant (100 μL) was transferred to a sampling vial for UPLC-QTOF-MS/MS analysis. Meanwhile, the 100 μL aliquot of each sample was also transferred to a sampling vial and mixed as a quality control (QC) to check the stability of the system and method.

#### 4.6.2. Metabolites Acquisition

An Agilent 6520 UPLC-QTOF MS/MS (Agilent Technologies, Santa Clara, CA, USA) was used in the study. Chromatographic separations were performed on an XSELECTTM HSS T3 column (2.1 mm × 100 mm, 2.5 μm, Waters, Milford, CT, USA) at a column oven temperature of 40 °C; 0.1% formic acid (A) and ACN (B) were used as the mobile phase. The gradient conditions were as follows: 0–2 min, 5% buffer B; 2–17 min, 5–95% buffer B; 17–20 min, 95% buffer B; 20–21 min, 95% buffer B. The post time was 6 min for column equilibration. The flow rate was maintained at 0.4 mL/min, the injection volume was 4 μL and the auto-sampler temperature was set at 4 °C. Electrospray ionization source (ESI) was set in both positive and negative-ion mode, and MS parameters were performed as follows: the scanning range was set 50~1100 *m*/*z*, electrospray capillary voltage with 4 kV in positive mode and 3.5 kV in negative mode were used, the nebulizer pressure was set at 45 psi, nitrogen was used as drying gas with a flow rate of 11 L/min and temperature was set as 350 °C, fragment voltage was maintained at 120 V, skimmer voltage was set at 60 V, Octopole RF Peake was set at 750 V, 121.0509 Da and 922.0098 Da were used at reference masses (*m*/*z*).

#### 4.6.3. Data Reduction and Pattern Recognitio

All data were acquired using Agilent MassHunter workstation software version B.01.04 (Agilent, Santa Clara, CA, USA). Firstly, the UPLC-QTOF MS/MS raw data were converted to mzdata files. The isotope interferences were excluded and the threshold of the absolute peak height was set at 500. The R package “xcms” was employed to generate a data matrix through peak extraction, alignment and integration, and the forma visual table including sample name, and peak indexes (*m*/*z*-Rt pairs and peak area). XCMS parameters were default settings except for the following: fwhm = 8, bw = 10 and snthersh = 5. All the ions were filtered based on the 80% rule before all of the detected ions in each sample were normalized to the sum of the peak area to obtain the relative intensity of metabolites based on MATLAB7.1 (MathWorks, Natick, MA, USA). After being normalized, ion intensities were converted to CSV (Comma-Separated Values) data and imported into the SIMCA-P program (version 12.0, Umetrics, Umea, Sweden) for principal component analysis (PCA) and orthogonal partial least-squares-discriminant analysis (OPLS-DA) after mean-centering and Pareto scaling. The parameters (R2X, R2Y, and Q2Y) showing the goodness of fit and prediction were assessed by SIMCA-P for internal validation.

In order to verify the different metabolites, first ions based on the extracted ion chromatogram (EIC) and then the extracted molecular weight with the common metabolite databases, such as the Human Metabolome Database, (http://metlin.scripps.edu) were confirmed. KEGG website was also adopted to enrich the relative pathways for these metabolites.

### 4.7. Statistics

All data were expressed as the mean ± standard deviation (SD). Statistically significant differences between groups were determined by one-way ANOVA and the Tukey test for multiple comparisons. All results were considered to be statistically significant at *p* < 0.05.

## 5. Conclusions

In this study, we have explored the stress-induced mucus secretion and its constituent composition in the jellyfish *A. coerulea* by a combination of proteomics and metabolomics. Our first conclusion is that two different but successive phases have been drawn from the initial stress to the final autolysis and death, with an obvious inflection point occurring at 30 min after removing the jellyfish from seawater. The results of proteomics using GO and KEGG analyses drew our second conclusion that the proteins in stress-induced mucus are independent (i.e., different) to those in tissue homogenate. We also identified that the mucus-enriched proteins are mainly located in the extracellular or membrane-associated region, while the tissue-enriched proteins are located in the intracellular compartment. The results of metabolomics are of particular interest; the potential neurotransmitter or neuromodulator, tryptamine, displays the maximal FC value of 7.8, a significantly elevated value among 16 other different metabolites in stress-induced mucus compared to those of tissue homogenate. This supports the hypothesis that a drastic nerve stress response, as well as a tempestuous release of neurotransmitters, occurs upon stress initiation. Finally, 11 metalloproteinases, four serine proteinase inhibitors, three SODs, three complements, and four toxin-related proteins were successfully assigned to function as self-protective components. In summary, our results provide a constituent profile of proteins and metabolites in stress-induced mucus and tissue homogenate of *A. coerulea*. This profile is, we believe, particularly important in equipping us with a better understanding of the process of stress-induced mucus secretion, as well as signaling the important role these self-protective components play in the innate immunity of jellyfish and in the ongoing discovery of novel bioactive compounds.

## Figures and Tables

**Table 1:** Metabolite difference between the secreted mucus and tissue homogenate.

Metabolite	FC	*p* Value	Related Pathway	Metabolite	FC	*p* Value	Related Pathway
Tryptamine	7.80	<0.0001	Tryptophan metabolism	Linoleyl linolenate	0.28	<0.0001	
4-Hydroxy-l-proline	1.26	<0.0001	Arginine and proline metabolism	Uridine	0.19	<0.0001	Pyrimidine metabolism
Citrulline	1.17	<0.0005	Arginine biosynthesis	l-Proline	0.13	<0.0001	Arginine and proline metabolism
l-Leucine	1.14	<0.0001	Valine, leucine and isoleucine metabolism	Inosine	0.12	<0.0001	Purine metabolism
3-(Phosphoacetylamido)-l-alanine	1.09	<0.0001	l-asparagine biosynthesis	Hypoxanthine	0.12	<0.0001	Purine metabolism
l-Threonine	0.88	0.0007	Valine, leucine and isoleucine biosynthesis	l-Valine	0.07	<0.0001	Valine, leucine and isoleucine metabolism
l-Glutamate	0.54	<0.0001	Arginine and proline metabolism	Guanosine	0.05	<0.0001	Purine metabolism
Succinylacetone	0.44	<0.0001	Tyrosine metabolism				

Note: FC values were obtained by comparing the mean concentration of each metabolite in mucus with that in tissue; FC value > 1 indicates a higher concentration in mucus, while the value < 1 indicates a lower concentration in mucus (*n* = 10). Metabolites analyzed based on MS/MS chromatograms.

**Table 2:** Summary of self-protective proteins enriched in mucus from the jellyfish *A. coerulea*.

Accession	Swissprot Annotation	Description	Matched Species	Identify (%)
Metalloproteases				
TRINITY_DN45838_c0_g1|m.27904	sp|Q20191	Zinc metalloproteinase nas-13	*Caenorhabditis elegans*	42.6
TRINITY_DN44124_c13_g11|m.23323	sp|Q20191	Zinc metalloproteinase nas-13	*Caenorhabditis elegans*	34.9
TRINITY_DN35212_c0_g1|m.10040	sp|P55115	Zinc metalloproteinase nas-15	*Caenorhabditis elegans*	30.8
TRINITY_DN45621_c1_g1|m.27292	sp|Q8N119	Matrix metalloproteinase-21	*Mus musculus*	34.9
TRINITY_DN42325_c7_g8|m.19392	sp|P51511	Matrix metalloproteinase-15	*Homo sapiens*	33.6
TRINITY_DN47471_c0_g1|m.30857	sp|Q9UKF2	ADAM30	*Homo sapiens*	45.1
TRINITY_DN45145_c0_g4|m.26001	sp|Q05910	ADAM8	*Mus musculus*	34.8
TRINITY_DN55675_c0_g2|m.32080	sp|O75077	ADAM23	*Homo sapiens*	31.1
TRINITY_DN46375_c2_g1|m.29588	sp|Q9UKP4	ADMTMS7	*Homo sapiens*	41.5
TRINITY_DN45686_c0_g1|m.27481	sp|Q9P2N4	ADMTMS9	*Homo sapiens*	33.2
TRINITY_DN44955_c0_g2|m.25457	sp|Q69Z28	ADMTMS16	*Mus musculus*	31.4
TRINITY_DN44955_c0_g1|m.25456	sp|Q9UKP5	ADMTMS6	*Homo sapiens*	30.6
Serine protease inhibitors				
TRINITY_DN43329_c0_g6|m.21485	sp|Q6J201	serine protease inhibitor	*Cyanea capillata*	56.4
TRINITY_DN45322_c11_g3|m.26476	sp|Q60854	Serpin B6	*Mus musculus*	37.5
TRINITY_DN9397_c0_g3|m.37796	sp|Q4R3G2	Serpin B6	*Macaca fascicularis*	38.8
TRINITY_DN29892_c0_g1|m.6537	sp|Q4R3G2	Serpin B6	*Macaca fascicularis*	36.6
Superoxide dismutase				
TRINITY_DN37669_c0_g1|m.12406	sp|Q8HXP2	Superoxide dismutase [Mn], mitochondrial	*Macaca mulatta*	75.3
TRINITY_DN36380_c0_g1|m.11103	sp|P11428	Superoxide dismutase [Cu-Zn] 2	*Zea mays*	65.8
TRINITY_DN37490_c0_g1|m.12223	sp|P24706	Superoxide dismutase [Cu-Zn]	*Onchocerca volvulus*	51.8
Complements				
TRINITY_DN45426_c0_g1|m.26742	sp|Q00685	Complement C3	*Lethenteron camtschaticum*	30.7
TRINITY_DN45758_c0_g1|m.27712	sp|Q00685	Complement C3	*Lethenteron camtschaticum*	25.9
TRINITY_DN43257_c1_g1|m.21313	sp|P81187	Complement factor B	*Bos taurus*	23.6

## Supplementary Materials

The following are available online at http://www.mdpi.com/1660-3397/16/9/341/s1, Table S1: Sequences of self-protective proteins.

## References

[1] Zoccarato L., Celussi M., Pallavicini A., Fonda Umani S. (2016). *Aurelia aurita* Ephyrae Reshape a Coastal Microbial Community. Front. Microbiol..

[2] Li R., Yu H., Xue W., Yue Y., Liu S., Xing R., Li P. (2014). Jellyfish venomics and venom gland transcriptomics analysis of *Stomolophus meleagris* to reveal the toxins associated with sting. J. Proteom..

[3] Wilcox C.L., Headlam J.L., Doyle T.K., Yanagihara A.A. (2017). Assessing the Efficacy of First-Aid Measures in *Physalia sp.* Envenomation, Using Solution- and Blood Agarose-Based Models. Toxins.

[4] Zhang L., He Q., Wang Q., Zhang B., Wang B., Xu F., Wang T., Xiao L., Zhang L. (2014). Intracellular Ca^2+^ overload induced by extracellular Ca^2+^ entry plays an important role in acute heart dysfunction by tentacle extract from the jellyfish *Cyanea capillata*. Cardiovasc. Toxicol..

[5] Lazcanopérez F., Arellano R.O., Garay E., Arreguínespinosa R., Sánchezrodríguez J. (2017). Electrophysiological activity of a neurotoxic fraction from the venom of box jellyfish *Carybdea marsupialis*. Comp. Biochem. Physiol. Part C Toxicol. Pharmacol..

[6] Sánchez-Rodríguez J., Torrens E., Segura-Puertas L. (2006). Partial purification and characterization of a novel neurotoxin and three cytolysins from box jellyfish (*Carybdea marsupialis*) nematocyst venom. Arch. Toxicol..

[7] Ruan Z., Liu G., Guo Y., Zhou Y., Wang Q., Chang Y., Wang B., Zheng J., Zhang L. (2014). First report of a thioredoxin homologue in jellyfish: Molecular cloning, expression and antioxidant activity of CcTrx1 from *Cyanea capillata*. PLoS ONE.

[8] Harada K., Maeda T., Hasegawa Y., Tokunaga T., Ogawa S., Fukuda K., Nagatsuka N., Nagao K., Ueno S. (2011). Antioxidant activity of the giant jellyfish *Nemopilema nomurai* measured by the oxygen radical absorbance capacity and hydroxyl radical averting capacity methods. Mol. Med. Rep..

[9] Cassoli J.S., Verano-Braga T., Oliveira J.S., Montandon G.G., Cologna C.T., Peigneur S., Pimenta A.M., Kjeldsen F., Roepstorff P., Tytgat J. (2013). The proteomic profile of Stichodactyla duerdeni secretion reveals the presence of a novel O-linked glycopeptide. J. Proteom..

[10] Stabili L., Schirosi R., Parisi M.G., Piraino S., Cammarata M. (2015). The Mucus of *Actinia equina* (Anthozoa, Cnidaria): An Unexplored Resource for Potential Applicative Purposes. Mar. Drugs.

[11] Provan F., Nilsen M.M., Larssen E., Uleberg K.E., Sydnes M.O., Lyng E., Oysaed K.B., Baussant T. (2016). An evaluation of coral lophelia pertusa mucus as an analytical matrix for environmental monitoring: A preliminary proteomic study. J. Toxicol. Environ. Health Part A.

[12] Salinas E.M., Cebada J., Valdes A., Garateix A., Aneiros A., Alvarez J.L. (1997). Effects of a toxin from the mucus of the Caribbean sea anemone (*Bunodosoma granulifera*) on the ionic currents of single ventricular mammalian cardiomyocytes. Toxicon.

[13] Patwa A., Thiéry A., Lombard F., Lilley M.K., Boisset C., Bramard J.F., Bottero J.Y., Barthélémy P. (2015). Accumulation of nanoparticles in “jellyfish” mucus: A bio-inspired route to decontamination of nano-waste. Sci. Rep..

[14] Hanaoka K.I., Ohno H., Wada N., Ueno S., Goessler W., Kuehnelt D., Schlagenhaufen C., Kaise T., Irgolic K.J. (2001). Occurrence of organo-arsenicals in jellyfishes and their mucus. Chemosphere.

[15] Patel D.M., Brinchmann M.F. (2017). Skin mucus proteins of lumpsucker (*Cyclopterus lumpus*). Biochem. Biophys. Rep..

[16] Li S., Jia Z., Li X., Geng X., Sun J. (2014). Calmodulin is a stress and immune response gene in Chinese mitten crab *Eriocheir sinensis*. Fish. Shellfish Immunol..

[17] Rajan B., Fernandes J.M., Caipang C.M., Kiron V., Rombout J.H., Brinchmann M.F. (2011). Proteome reference map of the skin mucus of Atlantic cod (*Gadus morhua*) revealing immune competent molecules. Fish. Shellfish Immunol..

[18] Elise H., Baptiste L., Ruddy W., Peter L. (2015). An integrated transcriptomic and proteomic analysis of sea star epidermal secretions identifies proteins involved in defense and adhesion. J. Proteom..

[19] Jatkar A.A., Brown B.E., Bythell J.C., Guppy R., Morris N.J., Pearson J.P. (2010). Coral mucus: The properties of its constituent mucins. Biomacromolecules.

[20] Shanks A.L., Graham W.M. (1988). Chemical defense in a scyphomedusa. Mar. Ecol. Prog. Ser..

[21] Heeger T., Möller H. (1987). Ultrastructural observations on prey capture and digestion in the scyphomedusa *Aurelia aurita*. Mar. Biol..

[22] Bonnet D., Molinero J.C., Schohn, Yahia M.D. (2012). Seasonal changes in the population dynamics of *Aurelia aurita* in Thau lagoon. Cah. De Biol. Mar..

[23] Scorrano S., Aglieri G., Boero F., Dawson M.N., Piraino S. (2016). Unmasking Aurelia species in the Mediterranean Sea: An integrative morphometric and molecular approach. Zool. J. Linn. Soc..

[24] Wang Y., Jiang H., Meng H., Li J., Yang X.J., Zhao B., Sun Y., Bao T. (2017). Antidepressant Mechanism Research of Acupuncture—Insights from an Genome-wide Transcriptome Analysis of Frontal Cortex in Rats with Chronic Restraint Stress. Evid. Based Complement. Altern. Med..

[25] Takeda S., Takeya H., Iwanaga S. (2012). Snake venom metalloproteinases: Structure, function and relevance to the mammalian ADAM/ADAMTS family proteins. Biochim. Biophys. Acta.

[26] Cristina S.M., Tamires L.S., Vieira S.M., Martins M.C., Maria S.F., Cortes F.K., Fábio O., Patriarca M.T.W., Roberto M.J. (2016). Interaction between TNF and BmooMP-Alpha-I, a Zinc Metalloprotease Derived fromBothrops moojeni Snake Venom, Promotes Direct Proteolysis of This Cytokine: Molecular Modeling and Docking at a Glance. Toxins.

[27] Jacob-Ferreira A.L., Menaldo D.L., Sartim M.A., Riul T.B., Dias-Baruffi M., Sampaio S.V. (2016). Antithrombotic activity of Batroxase, a metalloprotease from Bothrops atrox venom, in a model of venous thrombosis. Int. J. Biol. Macromol..

[28] Kang C., Han D.Y., Park K.I., Pyo M.J., Heo Y., Lee H., Kim G.S., Kim E. (2014). Characterization and neutralization of Nemopilema nomurai (Scyphozoa: Rhizostomeae) jellyfish venom using polyclonal antibody. Toxicon.

[29] Liu G., Zhou Y., Liu D., Wang Q., Ruan Z., He Q., Zhang L. (2015). Global Transcriptome Analysis of the Tentacle of the Jellyfish Cyanea capillata Using Deep Sequencing and Expressed Sequence Tags: Insight into the Toxin- and Degenerative Disease-Related Transcripts. PLoS ONE.

[30] Li N., Chiang D.Y., Wang S., Wang Q., Sun L., Voigt N., Respress J.L., Ather S., Skapura D.G., Jordan V.K. (2014). Ryanodine receptor-mediated calcium leak drives progressive development of an atrial fibrillation substrate in a transgenic mouse model. Circulation.

[31] Van Lint P., Libert C. (2007). Chemokine and cytokine processing by matrix metalloproteinases and its effect on leukocyte migration and inflammation. J. Leukocyte Biol..

[32] Brocker C.N., Vasiliou V., Nebert D.W. (2009). Evolutionary divergence and functions of the ADAM and ADAMTS gene families. Hum. Genom..

[33] Wolfsberg T.G., Straight P.D., Gerena R.L., Huovila A.P., Primakoff P., Myles D.G., White J.M. (1995). ADAM, a widely distributed and developmentally regulated gene family encoding membrane proteins with a disintegrin and metalloprotease domain. Dev. Biol..

[34] Apte S.S. (2004). A disintegrin-like and metalloprotease (reprolysin type) with thrombospondin type 1 motifs: The ADAMTS family. Int. J. Biochem. Cell. Biol..

[35] Kelwick R., Desanlis I., Wheeler G.N., Edwards D.R. (2015). The ADAMTS (A Disintegrin and Metalloproteinase with Thrombospondin motifs) family. Genome Biol..

[36] Rawlings N.D., Barrett A.J. (1995). Evolutionary families of metallopeptidases. Methods Enzymol..

[37] Manzetti S., McCulloch D.R., Herington A.C., van der Spoel D. (2003). Modeling of enzyme-substrate complexes for the metalloproteases MMP-3, ADAM-9 and ADAM-10. J. Comput. Aided Mol. Des..

[38] Hunt L.T., Dayhoff M.O. (1980). A surprising new protein superfamily containing ovalbumin, antithrombin-III, and alpha 1-proteinase inhibitor. Biochem. Biophys. Res. Commun..

[39] Rawlings N.D., Tolle D.P., Barrett A.J. (2004). Evolutionary families of peptidase inhibitors. Biochem. J..

[40] Morjen M., Kallechziri O., Bazaa A., Othman H., Mabrouk K., Zouarikessentini R., Sanz L., Calvete J.J., Srairiabid N., El Ayeb M. (2013). PIVL, a new serine protease inhibitor from Macrovipera lebetina transmediterranea venom, impairs motility of human glioblastoma cells. Matrix Biol. J. Int. Soc. Matrix Biol..

[41] Matsui T., Fujimura Y., Titani K. (2000). Snake venom proteases affecting hemostasis and thrombosis. Biochim. Biophys. Acta..

[42] Mourão C.B., Schwartz E.F. (2013). Protease Inhibitors from Marine Venomous Animals and Their Counterparts in Terrestrial Venomous Animals. Mar. Drugs.

[43] Zelko I.N., Mariani T.J., Folz R.J. (2002). Superoxide dismutase multigene family: A comparison of the CuZn-SOD (SOD1), Mn-SOD (SOD2), and EC-SOD (SOD3) gene structures, evolution, and expression. Free Radic Biol. Med..

[44] Kim K.Y., Sang Y.L., Cho Y.S., Bang I.C., Kim K.H., Dong S.K., Nam Y.K. (2007). Molecular characterization and mRNA expression during metal exposure and thermal stress of copper/zinc- and manganese-superoxide dismutases in disk abalone, Haliotis discus discus. Fish. Shellfish Immunol..

[45] Yang J., Dong S., Jiang Q., Si Q., Liu X., Yang J. (2013). Characterization and expression of cytoplasmic copper/zinc superoxide dismutase (CuZn SOD) gene under temperature and hydrogen peroxide (H_2_O_2_) in rotifer *Brachionus calyciflorus*. Gene.

[46] Umasuthan N., Bathige S.D., Revathy K.S., Lee Y., Whang I., Choi C.Y., Park H.C., Lee J. (2012). A manganese superoxide dismutase (MnSOD) from Ruditapes philippinarum: Comparative structural- and expressional-analysis with copper/zinc superoxide dismutase (Cu/ZnSOD) and biochemical analysis of its antioxidant activities. Fish. Shellfish Immunol..

[47] Broxton C.N., Culotta V.C. (2016). SOD Enzymes and Microbial Pathogens: Surviving the Oxidative Storm of Infection. PLoS Pathog..

[48] Lu X., Wang C., Liu B. (2015). The role of Cu/Zn-SOD and Mn-SOD in the immune response to oxidative stress and pathogen challenge in the clam Meretrix meretrix. Fish. Shellfish Immunol..

[49] Bakshani C.R., Morales-Garcia A.L., Althaus M., Wilcox M.D., Pearson J.P., Bythell J.C., Burgess J.G. (2018). Evolutionary conservation of the antimicrobial function of mucus: A first defence against infection. NPJ Biofilms Microbiomes.

[50] Abrams M.J., Goentoro L. (2016). Symmetrization in jellyfish: Reorganization to regain function, and not lost parts. Zoology (Jena).

[51] Johansson M.E., Sjovall H., Hansson G.C. (2013). The gastrointestinal mucus system in health and disease. Nat. Rev. Gastroenterol. Hepatol..

[52] Yang X., Steukers L., Forier K., Xiong R., Braeckmans K., Reeth K.V., Nauwynck H. (2014). A Beneficiary Role for Neuraminidase in Influenza Virus Penetration through the Respiratory Mucus. PLoS ONE.

[53] Hansen L.K., Becher N., Bastholm S., Glavind J., Ramsing M., Kim C.J., Romero R., Jensen J.S., Uldbjerg N. (2013). The cervical mucus plug inhibits, but does not block, the passage of ascending bacteria from the vagina during pregnancy. Acta Obstet. Gynecol Scand..

[54] Casado B., Pannell L.K., Iadarola P., Baraniuk J.N. (2010). Identification of human nasal mucous proteins using proteomics. Proteomics.

[55] Panicker G., Ye Y., Wang D., Unger E.R. (2010). Characterization of the Human Cervical Mucous Proteome. Clin. Proteom..

[56] De D.A., Idolo A., Bagordo F., Grassi T., Leomanni A., Serio F., Guido M., Canitano M., Zampardi S., Boero F. (2014). Impact of stinging jellyfish proliferations along south Italian coasts: Human health hazards, treatment and social costs. Int. J. Environ. Res. Public Health.

[57] Wang B., Zhang L., Zheng J., Wang Q., Wang T., Lu J., Wen X., Zhang B., Liu G., Zhang W. (2013). Multiple organ dysfunction: A delayed envenomation syndrome caused by tentacle extract from the jellyfish Cyanea capillata. Toxicon.

[58] Nagai H., Takuwa K., Nakao M., Ito E., Miyake M., Noda M., Nakajima T. (2000). Novel proteinaceous toxins from the box jellyfish (sea wasp) Carybdea rastoni. Biochem. Biophys. Res. Commun..

[59] Nagai H., Takuwa K., Nakao M., Sakamoto B., Crow G.L., Nakajima T. (2000). Isolation and characterization of a novel protein toxin from the Hawaiian box jellyfish (sea wasp) Carybdea alata. Biochem. Biophys. Res. Commun..

[60] Brinkman D., Burnell J. (2007). Identification, cloning and sequencing of two major venom proteins from the box jellyfish, *Chironex fleckeri*. Toxicon.

[61] Brinkman D.L., Konstantakopoulos N., McInerney B.V., Mulvenna J., Seymour J.E., Isbister G.K., Hodgson W.C. (2014). Chironex fleckeri (box jellyfish) venom proteins: Expansion of a cnidarian toxin family that elicits variable cytolytic and cardiovascular effects. J. Biol. Chem..

[62] Brinkman D.L., Burnell J.N. (2009). Biochemical and molecular characterisation of cubozoan protein toxins. Toxicon.

[63] Brinkman D.L., Jia X., Potriquet J., Kumar D., Dash D., Kvaskoff D., Mulvenna J. (2015). Transcriptome and venom proteome of the box jellyfish *Chironex fleckeri*. BMC Genom..

[64] Li R., Yu H., Yue Y., Liu S., Xing R., Chen X., Li P. (2016). Combined proteomics and transcriptomics identifies sting-related toxins of jellyfish *Cyanea nozakii*. J. Proteom..

[65] Ni Y., Xie G., Jia W. (2014). Metabonomics of human colorectal cancer: New approaches for early diagnosis and biomarker discovery. J. Proteome Res..

[66] Peng B., Li H., Peng X.X. (2015). Functional metabolomics: From biomarker discovery to metabolome reprogramming. Protein Cell.

[67] Quinones M.P., Kaddurah-Daouk R. (2009). Metabolomics tools for identifying biomarkers for neuropsychiatric diseases. Neurobiol. Dis..

[68] Lock J.Y., Carlson T.L., Carrier R.L. (2018). Mucus models to evaluate the diffusion of drugs and particles. Adv. Drug Deliv. Rev..

[69] Murgia X., Loretz B., Hartwig O., Hittinger M., Lehr C.M. (2018). The role of mucus on drug transport and its potential to affect therapeutic outcomes. Adv. Drug Deliv. Rev..

[70] Tittarelli R., Mannocchi G., Pantano F., Romolo F.S. (2015). Recreational Use, Analysis and Toxicity of Tryptamines. Curr. Neuropharmacol..

[71] Goddijn O.J., Lohman F.P., de Kam R.J., Schilperoort R.A., Hoge J.H. (1994). Nucleotide sequence of the tryptophan decarboxylase gene of *Catharanthus roseus* and expression of tdc-gusA gene fusions in *Nicotiana tabacum*. Mol. Gen. Genet..

[72] Guenter J., Lenartowski R. (2016). Molecular characteristic and physiological role of DOPA-decarboxylase. Postepy Higieny i Medycyny Doswiadczalnej.

[73] Brandt S.D., Freeman S., McGagh P., Abdul-Halim N., Alder J.F. (2004). An analytical perspective on favoured synthetic routes to the psychoactive tryptamines. J. Pharm. Biomed. Anal..

[74] Mohammad-Zadeh L.F., Moses L., Gwaltney-Brant S.M. (2008). Serotonin: A review. J. Vet. Pharmacol. Ther..

[75] Mayorova T.D., Kosevich I.A., Melekhova O.P. (2012). On some features of embryonic development and metamorphosis of Aurelia aurita (Cnidaria, Scyphozoa). Russ. J. Dev. Biol..

[76] Fossat P., Bacque-Cazenave J., De Deurwaerdere P., Delbecque J.P., Cattaert D. (2014). Comparative behavior. Anxiety-like behavior in crayfish is controlled by serotonin. Science.

[77] Zhao Y.T., Valdivia C.R., Gurrola G.B., Powers P.P., Willis B.C., Moss R.L., Jalife J., Valdivia H.H. (2015). Arrhythmogenesis in a catecholaminergic polymorphic ventricular tachycardia mutation that depresses ryanodine receptor function. Proc. Natl. Acad. Sci. USA.

[78] Da Silveira R.B., Wille A.C., Chaim O.M., Appel M.H., Silva D.T., Franco C.R., Toma L., Mangili O.C., Gremski W., Dietrich C.P. (2007). Identification, cloning, expression and functional characterization of an astacin-like metalloprotease toxin from *Loxosceles intermedia* (brown spider) venom. Biochem. J..

[79] Molehin A.J., Gobert G.N., McManus D.P. (2012). Serine protease inhibitors of parasitic helminths. Parasitology.

[80] Zhao Y.R., Xu Y.H., Jiang H.S., Xu S., Zhao X.F., Wang J.X. (2014). Antibacterial activity of serine protease inhibitor 1 from kuruma shrimp Marsupenaeus japonicus. Dev. Comp. Immunol..

[81] Alscher R.G., Erturk N., Heath L.S. (2002). Role of superoxide dismutases (SODs) in controlling oxidative stress in plants. J. Exp. Bot..

[82] Wang Y., Branicky R., Noe A., Hekimi S. (2018). Superoxide dismutases: Dual roles in controlling ROS damage and regulating ROS signaling. J. Cell. Biol..

[83] Poole A.Z., Kitchen S.A., Weis V.M. (2016). The Role of Complement in Cnidarian-Dinoflagellate Symbiosis and Immune Challenge in the Sea Anemone Aiptasia pallida. Front. Microbiol..

[84] Kimura A., Sakaguchi E., Nonaka M. (2009). Multi-component complement system of Cnidaria: C3, Bf, and MASP genes expressed in the endodermal tissues of a sea anemone, *Nematostella vectensis*. Immunobiology.

[85] Zhou Z., Sun D., Yang A., Dong Y., Chen Z., Wang X., Guan X., Jiang B., Wang B. (2011). Molecular characterization and expression analysis of a complement component 3 in the sea cucumber (*Apostichopus japonicus*). Fish. Shellfish Immunol..

